# Ontario Veterinary College

**Published:** 1891-01

**Authors:** 


### VETERINARY COLLEGE NOTES.

*Ontario Veterinary College.*—The Christmas examinations of this institution were closed on Friday, December the 19th. They were held in the examining hall of the new College on Temperance Street, Toronto, and were conducted in the usual vigorous and impartial manner. The Board of Examiners are appointed by the Agricultural and Arts Association of the Province of Ontario.

The Examining Board of this College ever since its inception in 1866 has been a duly recognized and properly appointed body of professional men. And the high standing which its graduates have attained are indications of the value of the teachings which they have received at the College; also of the vigorous nature and impartiality of the final examinations.

The following is the result :

### THE GRADUATING CLASS.

T. F. Arnold, Lewistown, Mo., U.S.; L. F. Baldock, Mount Charles, Ont.; J. H. Cornell, Lambeth, Ont. ; Newton Cossitt, Brockville, Ont.; D. Culham, Sheffield, Ont.; John M. Currie, Mitchell, Ont.; J. C. Faughnan, Bodines, Penn., U.S; W. J. Fleming, Millhaven, Ont.; James R. Frank, Strathroy, Ont.; R. G. Gilmour, Almonte, Ont.; James Harrison, Fergus, Ont.; George M. Hodgins, Lucan, Ont.; S. T. Holder, Bloomington, Ont.; James A. Kelley, Orono, Ont.; Samuel Kennedy, Rupert, Quebec; William Leslie, Northport, Ont.; Lyman D. Lockwood, Pennyan, N. Y., U. S.;

Adam McMillan, Carberry, Man.; Seth Mathers, Markdale, Ont.; W. D. Miller, Findlay, Ohio, U. S.; John F. Milne, Clinton, Ont.; A. Morren, Minesing, Ont.; R. J. Nelson, Paisley, Ont.; L. N. Patterson, Oakville, Ont.; C. L. Peck, Beamsville, Ohio, U. S.; Charles Pink, Ottawa, Ont.; W. H. Simon, St. John, New Brunswick; J. Steiner, Bergen, N. Y., U. S.; A. E. Taylor, Brampton, Ont.; John H. Teller, Simcoe, Ont.; N. Thomson, Toronto. Ont.; George N. N. Wilkins, Baysville, Muskoka, Ont.

PRIMARY EXAMINATIONS.

Frank Barnes, Materia Medica; D. Lewis, Materia Medica; John B. Sowers, Materia Medica.

